# The Current Practice of Screening, Prevention, and Treatment of Androgen-Deprivation-Therapy Induced Osteoporosis in Patients with Prostate Cancer

**DOI:** 10.1155/2012/958596

**Published:** 2012-04-30

**Authors:** Humaid O. Al-Shamsi, Arthur N. Lau, Kartika Malik, Abdulaziz Alamri, George Ioannidis, Tom Corbett, J. D. Adachi, Alexandra Papaioannou

**Affiliations:** ^1^Division of Medical Oncology, Department of Medicine, Juravinski Cancer Centre, McMaster University Hamilton Health Sciences, Hamilton, ON, Canada L8V 5C2; ^2^Division of Rheumatology, Department of Medicine, St. Joseph Hospital, McMaster University Hamilton Health Sciences, Hamilton, ON, Canada L8N 1Y2; ^3^Division of Geriatric Medicine, Department of Medicine, University of Toronto, Toronto, ON, Canada M5S 2E9; ^4^Division of Urology, Department of Surgery, St. Joseph Hospital, McMaster University Hamilton Health Sciences, Hamilton, ON, Canada L8N 1Y2; ^5^Department of Medicine, McMaster University Hamilton Health Sciences, Hamilton, ON, Canada L8S 4K1; ^6^Division of Radiation Oncology, Department of Oncology, Juravinski Cancer Centre, McMaster University Hamilton Health Sciences, Hamilton, ON, Canada L8V 5C2; ^7^Division of Medicine, Department of Medicine, McMaster University Hamilton Health Sciences, Hamilton, ON, Canada L8N 1Y2; ^8^Division of Geriatric Medicine, Department of Medicine, McMaster University Hamilton Health Sciences, Hamilton, ON, Canada L8S 4K1

## Abstract

*Introduction*. ADT is used in the management of locally advanced and metastatic disease. The detrimental effect of ADT on bone density is well documented. This study assesses care gaps in screening, prevention and treatment of osteoporosis among prostate cancer patients. *Methods*. We conducted a retrospective cohort study for patients diagnosed with non-metastatic prostate cancer on ADT. Charts from a tertiary oncology center were assessed for utilization of DXA scan, prescription of calcium, vitamin D, calcitonin and bisphosphonates.Bivariate analysis was used to determine the effect of patient characteristics and likelihood for osteoporosis screening. *Results*. 149 charts were reviewed, with 3-year mean follow-up. 58.8% of men received a baseline DXA, of which 20.3% had a repeat DXA within their follow-up periods.In all, 28% were appropriately screened and managed for osteoporosis (received repeat DXA, bisphosphonate). In bivariate analysis, the number of ADT injections which correlate with the duration of androgen suppression was significantly associated with the number of DXA scans. *Conclusions*. Our study found a care gap in the screening, prevention, and treatment of osteoporosis in this population. Patients receiving the most ADT injections were more likely to be screened. Our results suggest healthcare providers treating prostate cancer are insufficiently screening and treating this susceptible population. We suggest baseline measurement of BMD at the initiation of ADT with periodic reassessment during therapy.

## 1. Introduction

Prostate cancer is the most frequently diagnosed cancer and is the most common cancer to afflict Canadian men; around 25,500 men will be diagnosed with prostate cancer in 2011 in Canada alone [1], and aside from nonmelanoma skin cancer it is the most common cancer diagnosed in American men [1]. Rates of prostate cancer in men are comparable to rates of breast cancer in women and since 1995, the incidence of prostate cancer in both the United States and Canada have increased by 1 percent annually [1]. This is largely due to the aging of the population [1]. Androgen deprivation therapy (ADT) comprises of gonadotropin-releasing hormone agonists and is usually administered in a depot form. ADT remains the standard first-line therapy for metastatic prostate cancer. In addition to metastatic disease, ADT has also been shown to improve survival in patients with locally advanced or high-risk localized prostate cancer [2–4]. There is an increasing role for ADT in patients with localized prostate cancer and low-volume extracapsular disease, [3, 5], and for patients with a biochemical PSA recurrence [6].

In North American men with nonmetastatic prostate cancer, the rate of use of ADT has increased from 3.7% in 1991 to 31% in 1999 [7]. Approximately 50% of men with prostate cancer will receive ADT at some point after their diagnosis [8]. The prevalence of prostate cancer has been increasing, partly due to the increased use of prostate-specific antigen screening tests [9]. Therefore, the overall survival of patients with prostate cancer is very high, with one report estimating a 5-year survival of 98.1% [10]. Given the high survival rate, this makes the long-term adverse effects from ADT even more important.

ADT has a number of important adverse effects, most of which are a consequence of drug-induced hypogonadism. One of the most common and clinically significant adverse effects includes osteoporosis and increased risk of fractures [11]. This is due to ADT's effect on reducing circulating levels of estrogen and testosterone, which subsequently increases rates of bone resorption and impairs new bone formation [12, 13]. Several studies have shown that the maximal decline in BMD takes place in the first year of ADT treatment, with up to 10% decline in that first year [12, 14–16]. This BMD decline is evident even within months of starting ADT [12] and subsequently leads to an increase in rate of fractures [17, 18].

The objective of this study was to assess the management practices of radiation oncologists who prescribe ADT for the management of early prostate cancer and assess if a care gap exists between current recommendations, as well as what is seen in practice, also identify factors, which increased the likelihood for radiation oncologists to screen men on ADT for osteoporosis.

## 2. Methods

### 2.1. Population

Patients treated with Androgen deprivation therapy followed at the Juravinski Cancer Centre in Hamilton, Ontario, Canada, were assessed between years 2008 and 2009. This is a tertiary referral center with 2000 prostate cancer patients referred per year for all stages of prostate cancer. All patients diagnosed with nonmetastatic prostate cancer confirmed by biopsy were screened, and ADT treatment at any point during their prostate cancer management was included in the study.

### 2.2. Study Design and Data Collection

Approval from the Hamilton Health Sciences Center's Ethics board was obtained. All the patients treated at the Juravinski Cancer Centre (JCC) were identified by hormonal treatment billing number. There were a total of 745 patient charts, and every fifth chart was reviewed in its entirety from the patient's initial clinic visit to the date of the audit. The duration of patient followup ranged from 6 months to 18 years, with the majority of patients followed up for more than 3 years.

The data collected include patient age, date of prostate cancer diagnosis, and age at which the diagnosis was made, and clinical prostate cancer data including the Gleason score, tumor stage, last PSA value, and the absence or presence of bony metastases. The presence of known risk factors for osteoporosis including prior fracture, corticosteroids use or hyperthyroidism, diabetes, smoking, and alcohol use phenytoin use, were also abstracted.

 Furthermore, the presence or absence of osteoporosis screening either before the initiation of ADT or during the followup period was abstracted. This was characterized by the performance of baseline Dual X-ray Absorptiometry (DXA) scans if ADT was planned for more than 6 months and repeat DXA scan at any point during the patient's followup for both normal and abnormal baseline DXA scans. The prescription of pharmacologic interventions was also abstracted including, calcium and vitamin D supplementation, oral or intravenous bisphosphonates, and calcitonin therapy [8].

Bivariate analysis was used to determine which of the above factors was more likely to prompt physicians to screen for osteoporosis. The factors assessed by bivariate analysis included the number of ADT injections, patient age at diagnosis, Gleason score, and PSA score at time of diagnosis.

## 3. Results

The charts of 149 men with nonmetastatic prostate cancer were reviewed. Demographics are presented in Table 1. The mean age of men was 73.3 years (SD = 7.4). The mean follow-up duration between diagnosis to time of data extraction was 4.3 years (SD = 0.24), and these men received a mean of 8.9 ADT (SD = 6.15) injections during their follow-up period (Table 1). Risk factors for osteoporosis included 49.3% who had a history of smoking, of which 16.7% were current smokers. In addition, 3.3% of men had a history of corticosteroid use, and 2% had a history of hyperthyroidism. A history of alcohol abuse was reported in 13.3% of men; the history of abuse was clearly documented in the chart; we could not verify the exact definition of abuse between different treating radiation oncologists. Five had a fracture prior to treatment with ADT; these include one hip fractures.

A total of 58.8% (*n* = 87) of men on ADTs received a baseline DXA scan, of which 20.3 (*n* = 17) had a repeat DXA scan at any point within their individual follow-up periods (Table 2). Bisphosphonates, calcium, vitamin D, and calcitonin were prescribed to 12.7, 35.6, 36.9%, and 1.3% of men respectively. Only 28% (42/148) of patients received both a baseline BMD and a followup BMD at some point during their followup or were started on a bisphosphonate (Table 2).

Of the 87 patients who received an initial screening BMD, 12 patients had a BMD in the osteoporosis range ≥2.5 and 13 patients in the osteopenia range (−2, 5< *t*-score <−1) (Table 3). Of the 12 patients with an initial BMD in the osteoporotic range, 3/12 (25%) patients were on a bisphosphonate, and 6/12 (50%) were on calcium supplementation, 6/12 (50%) were on vitamin D supplementation. In addition, only 4/12 (25%) received a repeat BMD at any time during the follow-up period. Of the 13 patients with an initial BMD in the osteopenia range, 4/13 (25%) patients were on a bisphosphonate, and 8/13 (50%) were on calcium supplementation, 8/13 (50%) were on vitamin D supplementation. Only 5/13 (25%) received a repeat BMD at any time during the follow-up period.

Using bivariate analysis, we also assessed if there were any prognostic factors which increased the likelihood for screening for osteoporosis (Table 4). The number of ADT injections increased the likelihood for being screened. The mean number of ADT injections in patients who received a follow-up DXA or were prescribed a bisphosphonate was 13.1 versus 7.9 (*P* ≤ 0.0001). Age, Gleason score, and PSA at the initial visit were not associated with increased screening.

## 4. Discussion

Androgen-deprivation therapy (ADT) in men with prostate cancer affects bone metabolism and is associated with a decrease in bone mineral density (BMD). The clinical implications of this bone loss have been well recognized, and managing skeletal health in this population is an emerging challenge.

To prevent osteoporosis in men on ADT, clinicians have been advised to screen patients for osteoporosis prior to the initiation of ADT with DXA scan [19, 20]. In addition, lifestyle modifications including smoking cessation, moderating alcohol intake, and regular exercise are encouraged [21, 22]. Pharmacological interventions, such as calcium, vitamin D, are recommended for particular individuals [19, 20, 23–25]. Bisphosphonates are also recommended for particular individuals at high risk of fractures. Denosumab is a newer agent, which has been approved recently by the FDA [26]. It is a fully human monoclonal antibody against receptor activator of nuclear factor-kappa B ligand which was also associated with increased bone mineral density at all sites and a reduction in the incidence of new vertebral fractures among men receiving androgen-deprivation therapy for nonmetastatic prostate cancer [27]. Despite these recommendations, few clinicians order a baseline DXA scan or prescribe bisphosphonates to patients undergoing ADT [28]. Several retrospective studies have assessed the actual management of bone health compared to current recommendations for monitoring and treatment, and these studies have shown that a care gap indeed exists in this population [29–31].

As a result of the potential consequences of ADT, expert guideline recommendations advocate for assessing men prescribed ADT for osteoporosis and to estimate the baseline fracture risk using an assessment tool, such as the World Health Organization fracture risk assessment tool [6, 21, 32, 33]. It is also recommended that these patients should receive a baseline DXA scan prior to initiating treatment [6, 21]. In addition, calcium and vitamin D supplementation are recommended to all men before starting ADT [34, 35]. Although bisphosphonates are not recommended for all patients, they are for patients with documented osteoporosis on DXA or a history of fractures [19, 36]. Bisphosphonates have been shown in a number of studies to be effective in improving bone mineral density in men on ADT [14, 37, 38]. Alternatives to bisphosphonates, including selective estrogen receptor modulators and denosumab, have also been shown to modestly increase in BMD of the hip and lumbar spine in men on ADT [39, 40].

In this study, 149 patients from the Juravinski Cancer Centre with nonmetastatic prostate cancer on ADT were assessed. From the 149 patients, only 58.8% received a baseline DXA scan to screen for osteoporosis prior to starting ADT. Only 20.3% of patients received a repeat DXA scan at any point during their follow-up period.

Although it is recommended to all patients on ADT, only 35.6% and 36.9% were prescribed calcium and vitamin D supplementation, respectively. There is no general agreement about whether or not men who are on a hormone blockade (ADT) to treat prostate cancer should be taking calcium and vitamin D supplements; however, there does seem to be some consensus that they should.

Given that there are geographic and seasonal variations in vitamin D, levels the issue becomes even more unclear. However, men in North American and Western Europe are at a higher risk for having low vitamin D levels so calcium and vitamin D are especially important in men receiving bisphosphonates or denosumab [41]. In terms of antiresorptive treatments, 12.7% of patients were prescribed a bisphosphonate and 1.3% prescribed calcitonin. We also considered less stringent criteria for defining screening and management of osteoporosis in our population, where we considered patient receiving a baseline DXA, follow-up DXA at during their follow-up period, or were treated with an anti-resorptive agent as being screened and managed appropriately. Although this is still not the optimal management for this high-risk population, only 28% of patients met these less stringent criteria we defined. Adding to the evidence that a care gap exists in this population, of the patients with an initial BMD in the osteoporosis range, only 25% were on a bisphosphonate, and only 50% were on calcium and vitamin D supplementations, respectively. Furthermore, in all of the charts we assessed, we found that none of the patients had their fracture risk assessed using one of the available fracture risk assessment tools (i.e., FRAX) [33, 42–44].

The results we report are quite alarming since this population of men are at particularly high risk for developing osteoporosis and nonpathological fractures [17]. This group of patients also received a relatively high number of ADT, a mean of 8.9 treatments per patient, which further increases their risk for future fractures [29]. In addition, the patients we assessed also have other risk factors for osteoporosis or fractures, including a history of smoking glucocorticoid use, and prescription of SSRIs or anticonvulsants.

At our institution, a definite care gap exists between the recommended care for these patients, and what is seen in clinical practice. The low rates of screening for and treatment of osteoporosis are similar to the rates reported by a group in New Mexico, who assessed the rates of screening, prevention, or treatment of osteoporosis in patients with nonmetastatic prostate cancer on ADT [31]. In that study, only 13% of patients received DXA scans, 21% of patients were on treatment with an oral or IV bisphosphonates, and 16% and 10% of patients were prescribed calcium and vitamin D supplementation, respectively [31]. In another study which included patients with metastatic prostate cancer, only 14.7% of patients received a DXA scan within the previous 3 years, or treatment with bisphosphonates, calcitonin, or estrogen within the past year, or supplementation with calcium and vitamin D [29]. These other studies, in conjunction with our findings, suggest that the lack of screening and treatment of osteoporosis in these patients is not merely a problem at the institutional level, but likely much more widespread. This remains a significant problem given the high incidence of prostate cancer and the common use of ADT for its management.

It is unclear why prostate cancer patients on ADT were rarely screened for osteoporosis and recommended to start calcium and vitamin D supplementation. These preventative measures are recommended to all patients prior to starting ADT.

It is also unclear why so few patients were prescribed an antiresorptive agent for the treatment of osteoporosis. It is likely a multifactorial issue leading to this care gap.

Using bivariate analysis, we determined that physicians were more likely to screen for osteoporosis in patients with a greater number of ADTs received. However, a decline in BMD is evident even within months of starting ADT. We hypothesize that a potential barrier to screening and treatment of osteoporosis is due to a lack of education of the oncologists and front-line staff regarding the impact of ADT on metabolic bone disease and its potential devastating consequences. Another area where further education and training may be required is the education of caregivers on the usefulness and application of a fracture risk assessment tool to predict those at high risk of future fractures (i.e., FRAX). This is quite evident as none of the patients we assessed had a fracture risk assessment using any of the available tools.

Future research should emphasize the implementation of the above-mentioned education initiatives for radiation oncologists and nurses practitioners who treat prostate cancer and assess if these education initiatives have any impact on the screening practices in this group. Several years ago, our group conducted a study assessing the use of educational protocols to improve the knowledge of family physicians in regards to evidence-based osteoporosis management and fracture risk factors [45]. After 1 year, the family physician's awareness of their patient's risk factors increased, and the utilization of bone mineral density testing in the high risk fracture group significantly increased as well. A similar system can be implemented for oncology practices.

Some limitations of our study included that the study was retrospective, and only a single cancer center was assessed. Also since this was a retrospective chart review, comprehensive documentation was required, so that data abstracted from the chart may not fully reflect the clinical care that patients receive. Unless specifically documented, we were not able to determine why patients were not screened for osteoporosis, and if patients received council regarding nonpharmacological recommendations to reduce falls and fractures. In addition, since vitamin D and calcium supplementation can be bought over the counter, these rates may be an under estimation as the true rates may not be captured through the chart review process. On the other hand, adherence to calcium and vitamin D is in general poor and confirmation of adherence was not possible.

## 5. Conclusions

Our study found a suboptimal rate of osteoporosis screening and preventative measures in men with nonmetastatic prostate cancer on ADT. These results are concerning given the high risk of this population to develop future osteoporosis and nonfragility fractures. The barriers leading to the care gap in this susceptible population remain to be determined. We hypothesize a lack of education regarding the actual degree of impact ADT has on bone metabolism, and the availability of tools to identify individuals at high risk of fractures are important factors that contribute to the care gap. Future research should focus on determining the specific barriers to screening and treatment of osteoporosis and assessing different strategies to counteract these barriers.

## Figures and Tables

**Table 1:** Baseline characteristics of all patients, including collected prevalence of osteoporosis risk factors.

Age (*n* = 149) (Standard Deviation)	73.3 years (7.4)
Years between diagnosis and study entry (*n* = 117)	4.3 years (0.2)
Number of ADT injections received (*n* = 148)	8.9 (6.1)
PSA at first visit (*n* = 150)	22.4 (61.5)
Gleason score (*n* = 149)	7.6 (1.0)
Height (*n* = 147)	170.7 cm (21.2)
Weight (*n* = 150)	88.2 kg (17.4)
Prostate cancer stage 1A, 1B, or 1C	24.6% (35/142)
Prostate cancer stage 2A, 2B, or 2C	44.4% (63/142)
Prostate cancer stage 3A, 3B, or 3C	31.0% (44/142)
Prior hip fracture before treatment	0% (0/147)
Prior vertebral fracture before treatment	1.4% (2/148)
Prior fracture before treatment (excluding hip or vertebral)	1.4% (2/148)
History of smoking	50.3% (74/147)
Current smoker at first visit	17% (25/147)
Steroid use during followup	3.3% (5/150)
SSRI use during followup	10.7% (16/150)
Anticonvulsant use during followup	4.7% (7/150)
HRT use during followup	13.5% (20/148)

**Table 2:** Evaluations screening strategies, prevention measures, and pharmacological treatment of osteoporosis.

Patient with BMD at baseline visit	58.8% (87/148)
Patient with BMD at any followup visit	20.3% (30/148)
Vitamin D supplementation during followup	36.9% (55/149)
Calcium supplementation during followup	35.6% (53/149)
Bisphosphonate use during followup	12.7% (18/142)
Calcitonin use during followup	0% (0/148)
Either a repeat BMD or treatment with a bisphosphonate during followup	28.4% (42/148)

**Table 3:** Of the 87 patients with a baseline BMD, 12 patients had a BMD value in the osteoporosis range and 13 in the osteopenia range. Percentage of patients on bisphosphonates, and calcium supplementation, vitamin D supplementation, calcitonin prescription was assessed based on baseline BMD values.

	Bisphosphonate prescription	Calcium supplementation	Vitamin D supplementation	Calcitonin prescription	Repeat BMD at followup
Baseline BMD in osteoporosis range	25% (3/12)	50% (6/12)	50% (6/12)	0% (0/12)	25% (4/12)
Baseline BMD in osteopenia range	4/13	8/13	8/13	0% (0/13)	5/13

**Table 4:** Bivariate analysis was used to determine which of the above factors was more likely to prompt physicians to screen for osteoporosis.

	Odds ratio	95% CI
Number of ADT treatments	1.24	1.096–1.403
Age	0.73	0.911–1.040
Gleason score	1.082	0.687–1.703
Years since diagnosis	1.109	0.915–1.345
Smoking history	1.43	0.565–3.621
Stage 1 A or B or C versus Stage 3 A or B or C	0.959	0.264–3.484
Stage 2 A or B or C versus Stage 3 A or B or C	1.61	0.539–4.808
