# Corrigendum to “The Current Practice of Screening, Prevention, and Treatment of Androgen-Deprivation-Therapy Induced Osteoporosis in Patients with Prostate Cancer”

**DOI:** 10.1155/2017/3432604

**Published:** 2017-12-03

**Authors:** Humaid O. Al-Shamsi, Arthur N. Lau, Kartika Malik, Abdulaziz Alamri, George Ioannidis, Tom Corbett, J. D. Adachi, Alexandra Papaioannou

**Affiliations:** ^1^Division of Medical Oncology, Department of Medicine, Juravinski Cancer Centre, McMaster University Hamilton Health Sciences, Hamilton, ON, Canada L8V 5C2; ^2^Division of Rheumatology, Department of Medicine, St. Joseph Hospital, McMaster University Hamilton Health Sciences, Hamilton, ON, Canada L8N 1Y2; ^3^Division of Geriatric Medicine, Department of Medicine, University of Toronto, Toronto, ON, Canada M5S 2E9; ^4^Division of Urology, Department of Surgery, St. Joseph Hospital, McMaster University Hamilton Health Sciences, Hamilton, ON, Canada L8N 1Y2; ^5^Department of Medicine, McMaster University Hamilton Health Sciences, Hamilton, ON, Canada L8S 4K1; ^6^Division of Radiation Oncology, Department of Oncology, Juravinski Cancer Centre, McMaster University Hamilton Health Sciences, Hamilton, ON, Canada L8V 5C2; ^7^Division of Medicine, Department of Medicine, McMaster University Hamilton Health Sciences, Hamilton, ON, Canada L8N 1Y2; ^8^Division of Geriatric Medicine, Department of Medicine, McMaster University Hamilton Health Sciences, Hamilton, ON, Canada L8S 4K1

In the article titled “The Current Practice of Screening, Prevention, and Treatment of Androgen-Deprivation-Therapy Induced Osteoporosis in Patients with Prostate Cancer” [1], there was an error regarding the FRAX® tool, which should be clarified as follows.

The article notes: “As a result of the potential consequences of ADT, expert guideline recommendations advocate for assessing men prescribed ADT for osteoporosis and to estimate the baseline fracture risk using an assessment tool, such as the World Health Organization fracture risk assessment tool [6, 21, 32, 33]”. However, the World Health Organization (WHO) did not develop, test, or endorse the FRAX® tool or its recommendations [2]. The metabolic bone disease unit at the University of Sheffield that developed FRAX® was a WHO Collaborating Centre from 1991 to 2010, but treatment guidelines must undergo a formal process before they can be endorsed by the WHO.
